# Nanocarriers Made of Natural Fatty Acids: Modulation of Their Release Profiles through Photo‐Crosslinking

**DOI:** 10.1002/anie.202415671

**Published:** 2024-12-04

**Authors:** Dong Zhang, Yuxuan Meng, Min Hao, Younan Xia

**Affiliations:** ^1^ The Wallace H. Coulter Department of Biomedical Engineering Georgia Institute of Technology and Emory University Atlanta GA 30332 USA; ^2^ School of Chemistry and Biochemistry Georgia Institute of Technology Atlanta GA 30332 USA

**Keywords:** Natural fatty acids, controlled release, drug delivery, carriers, photo-crosslinking

## Abstract

Natural fatty acids are attractive carrier materials for drug delivery, but their rapid dissolution and degradation in vivo calls for new strategies to enhance their stability. Here we report a simple and versatile method capable of photo‐crosslinking carriers made of natural fatty acids for drug delivery under controlled release. By optimizing the crosslinking density, the nanoscale carriers show a high drug loading efficiency, together with a stable network structure for minimal degradation in a body fluid mimic. Fluorescence microscopy analysis also reveals the exceptional intracellular stability of the crosslinked network, resulting in negligible cytotoxicity toward A549 cells up to 24 h when loaded with a potent anticancer drug. We further extend this strategy to microscale carriers fabricated using electrospray. Upon photo‐crosslinking, the carriers show a retarded release of nerve growth factor, resulting in slower neurite outgrowth from dorsal root ganglion. This work holds promise for addressing the efficacy and safety issues critical to nanomedicine and related applications.

## Graphical abstract

**Slow down**: Photo‐crosslinking is used to mitigate the dissolution and degradation of natural fatty acids in a body fluid mimic, achieving sustained release in cells with controllable kinetics for drug delivery, nanomedicine, and related applications.

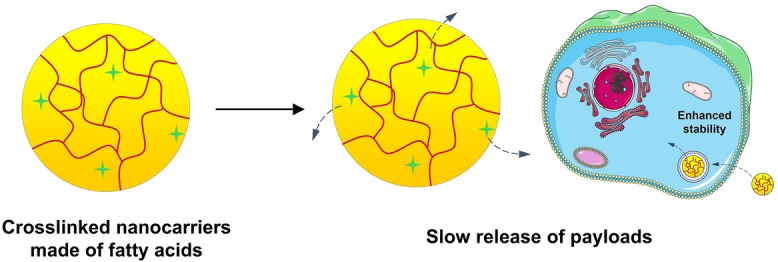

## Introduction

Natural fatty acids are indispensable for cellular energy production and a variety of metabolic pathways, underpinning many functions and processes vital to health and normal physiological development.[^1^, ^2^] A crucial role of fatty acids is to serve as substrates for the synthesis of adenosine triphosphate (ATP).^[3]^ To fulfill this role, the solubility of fatty acids significantly increases upon binding to plasma albumin and the dissolved molecules then break down to generate acetyl‐CoA through the beta‐oxidation process.^[4]^ While this increase in solubility offers metabolic advantages, formulating them into carriers for controlled release and drug delivery poses challenges as the rapid dissolution and degradation will result in premature or burst release.^[5]^ To slow down the degradation of carriers made of natural fatty acids, two major strategies have been explored in the literature: *i*) encapsulation in biocompatible and biodegradable polymers such as poly(lactic‐*co*‐glycolic acid) or polycaprolactone^[6]^ and *ii*) chemical modification by incorporating bulky groups or altering the chain structure (e.g., esterification or amidation).^[7]^ Encapsulation provides excellent protection for the active ingredients while extending their release up to several weeks. However, achieving a specific release profile can be challenging due to factors such as the degradation kinetics of the encapsulating material, variations in the environmental condition, and the difficulty in fine‐tuning the encapsulation process itself. On the other hand, chemical modification offers enhanced stability and even new functionality, but it is often costly and potentially introduces toxicity or undesirable side effects in vivo.[^8^, ^9^] More importantly, simple chemical modification alone typically cannot achieve sustained release of a payload over weeks or months.

Giving the structural diversity of fatty acids, such as the variations in chain length, degree of unsaturation (i.e., the number and position of double bonds), and inclusion of additional functional groups,[^10^, ^11^] creating a three‐dimensional network in the carrier particles made of fatty acids offers an effective route to modulate the release kinetics. An increasingly‐crosslinked network will resist degradation more effectively, ensuring prolonged shelf‐life and maintaining therapeutic efficacy or functional integrity over a longer time.^[12]^ In recent years, polyunsaturated fatty acids (PUFAs) have garnered considerable interest owing to their natural occurrence, biocompatibility, and the presence of relatively reactive double bonds.[^13^, ^14^] These features make them promising candidates for controlled release and related applications. However, while many PUFAs possess reactive double bonds to theoretically allow for photo‐crosslinking, not all PUFAs can be crosslinked under practical conditions. The crosslinking capability of a specific PUFA depends on its structural features, including the availability of reactive sites, the compatibility with initiators, and the reaction conditions used.[^15^, ^16^] For example, most unsaturated fatty acids, such as docosahexaenoic acid and linoleic acid, feature isolated double bonds and bulky alkyl chains in their molecular structures, making them low in reactivity toward photo‐crosslinking. When exposed to ultraviolet (UV) light in the absence of an appropriate stabilizer (e.g., tocopherol or ascorbic acid) or protective measures, these fatty acids are susceptible to aging or degradation rather than covalent crosslinking.[^17^, ^18^] Moreover, when used as phase change materials (PCMs), the presence of double bonds along the backbone of PUFAs introduces kinks and bends in the carbon chain, making it difficult to maintain them in the solid state at or above room temperature.[^19^, ^20^, ^21^] This characteristic may result in undesired burst release of the encapsulated drug from nanoscale carriers made of PUFAs when subjected to the physiological temperature.

Fascinated by the prior success in crosslinking nanoscale carriers (e.g., nanogels, micelles, liposomes, and polymersomes), this study aims to manipulate the release kinetics of carriers made of natural fatty acids by tuning the extent of crosslinking. We focus on mixtures containing two or more fatty acids for their enhanced drug loading capacity due to modified crystallization behavior, ability to maintain structural stability, and adjustable melting points to meet the requirements of specific applications. Using highly reactive conjugated linoleic acids (CLAs) as a model system, we demonstrate a simple and versatile method for controlling the crosslinking density via UV irradiation under mild conditions. We also establish a correlation between the crosslinking density and the degradation rate in vitro to achieve effective regulation of the release kinetics of different payloads. Benefiting from the enhanced robustness of a crosslinked network, the DOX‐loaded nanoscale carriers show excellent cellular stability and low cytotoxicity, maintaining minimal DOX release for at least 24 h. Moreover, the universal nature of the crosslinking strategy allows us to extend its application to other particulate systems, such as electrosprayed microparticles comprised of fatty acids. This adaptability across various formulations of fatty acids highlights its potential to advance applications in sustained release, offering promising prospects in an array of biological and biomedical applications.

## Results and Discussion

### Photo‐crosslinking of nanoscale carriers made of natural fatty acids

**Figure 1:**
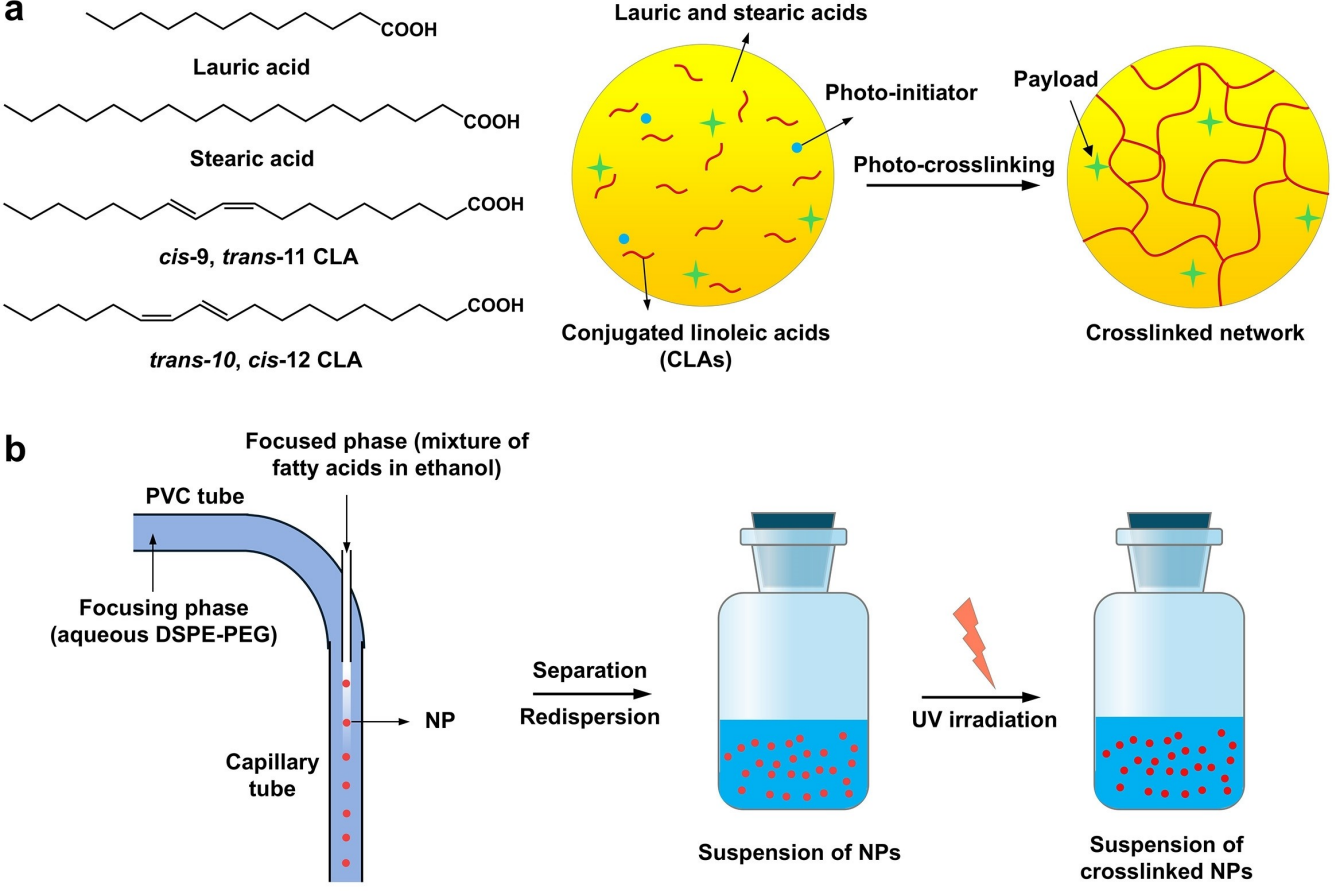
Illustrations showing the fabrication and subsequent photo‐crosslinking of nanoscale carriers composed of natural fatty acids. (a) Schematic of the procedure used for crosslinking NPs consisting of a mixture of lauric acid, steric acid, and conjugated linoleic acids (i.e., *cis*‐9, *trans*‐11 and *trans*‐10, *cis*‐12), along with 2,2‐dimethoxy‐2‐phenylacetophenone as a photo‐initiator. The formulation may also include 1‐pyrenedecanoic acid as a fluorescent probe for monitoring the degradation profile. (b) Schematic of a homemade fluidic device used for preparing the NPs. As the mixture of fatty acids in ethanol and a lipid solution in water meet and mix along the flow, the fatty acids precipitate out as uniform NPs at the interface between the focusing and focused phases. The NPs are purified and then subjected to UV irradiation to induce crosslinking.

We first formulated a cross‐linkable mixture that contained lauric acid and stearic acid at a mass ratio of 1 : 5, together with CLAs and a photo‐initiator (Figure 1a). Using a combination of hydrodynamic flow focusing in a fluidic device and anti‐solvent‐induced precipitation,^[22]^ the mixture was processed as nanoparticles (NPs) uniform in size. In this approach, hydrodynamic flow focusing was achieved by coaxially introducing the mixture in ethanol and an aqueous lipid solution (the anti‐solvent) as the focused and focusing phases, respectively (Figure 1b). When these two phases interacted at the interface, lipid‐capped NPs formed and precipitated out. The collected NPs then underwent purification through centrifugation, followed by exposure to UV irradiation for different periods to produce crosslinked products.

**Figure 2:**
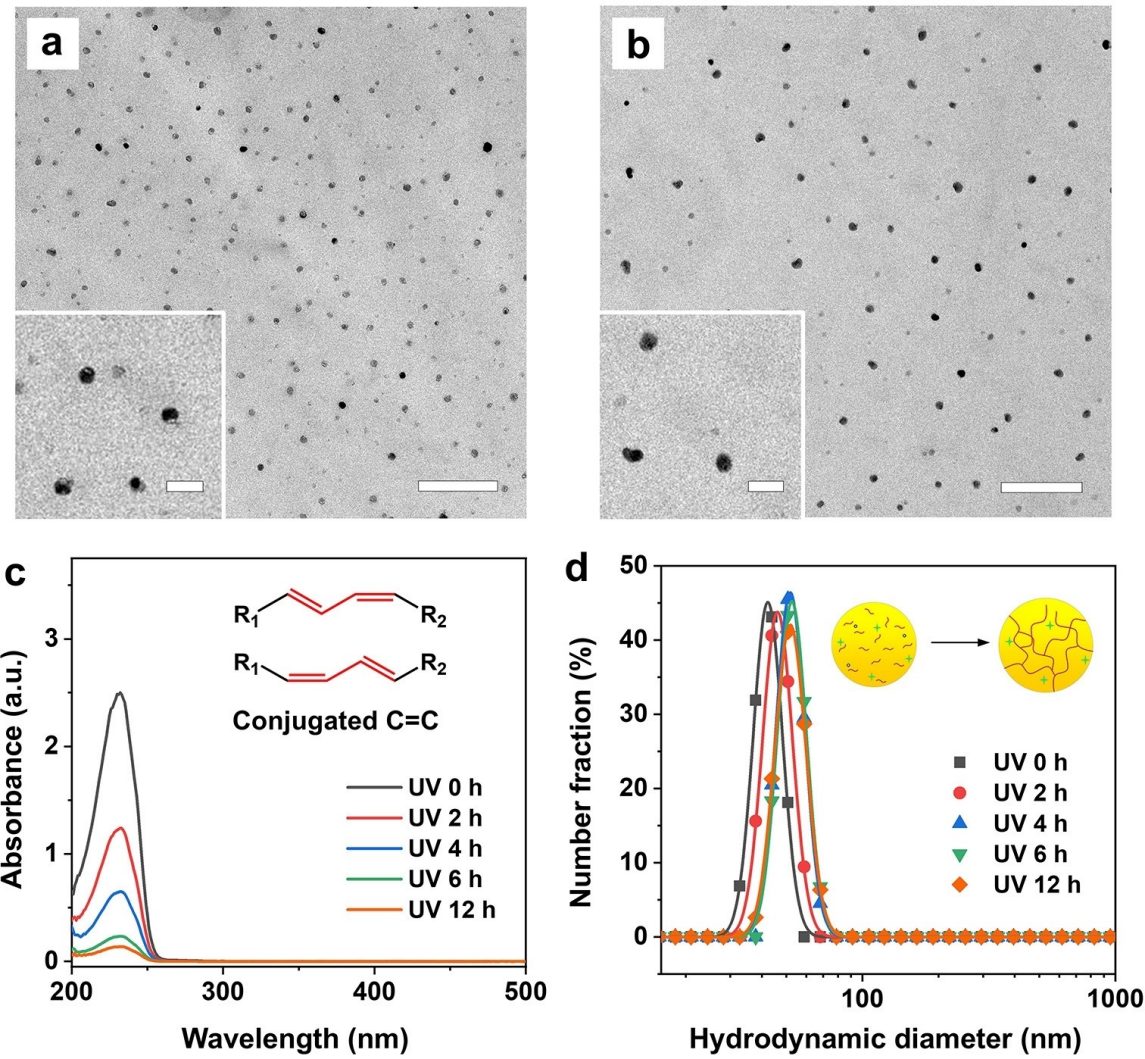
Characterizations of the NPs before and after UV‐crosslinking. (a, b) TEM images of the NPs before and after UV irradiation for 10 h. The scale bars in the panels and insets are 500 and 100 nm, respectively. (c) UV/Vis absorbance spectra of the mixture of fatty acids in ethanol before and after UV irradiation for 2, 4, 6, and 12 h. The non‐crosslinked and crosslinked NPs (10 μL, *ca*. 0.12 mg/mL) were dissolved in 1 mL of ethanol before testing. (d) Size distributions of the NPs, before and after UV exposure for 2, 4, 6, and 12 h, measured using DLS.

Figure 2a, b shows representative transmission electron microscopy (TEM) images of the NPs before and after UV‐crosslinking, respectively. At first glance, the NPs maintained a consistent diameter of about 40 nm after UV irradiation for 10 h. This result underscores that low‐power UV irradiation selectively promoted the formation of covalent bonds at the molecular level, leaving the macroscopic morphology of the NP intact. In general, π‐conjugated systems exhibit unique electronic structures capable of absorbing light in the UV and/or visible (vis) regions of the electromagnetic spectrum.^[23]^ By analyzing the absorption spectrum in the UV/Vis regions and, in particular, monitoring the peaks and patterns corresponding to the electronic transitions, one can infer the presence and abundance of the conjugated double bonds in a system. Figure 2c shows the UV/Vis spectra recorded from aqueous suspensions of the NPs before and after UV crosslinking. As the UV exposure time was increased from 0 to 12 h, the intensity of the characteristic absorption peak at 232 nm, corresponding to the π‐conjugated system, dropped by 94.5 %. This observation implies progressive depletion of CLAs over time due to their gradual conversion into C−C bonds with the increase in crosslinking density. Figure S1a shows the Fourier‐transform infrared (FTIR) spectra recorded from dry samples of the NPs before and after UV crosslinking for 0–12 h. From the spectra, we only observed a minor difference for the peak at 2950 cm^−1^ before and after crosslinking because this peak corresponds to the symmetrical stretching of C−H bonds in both alkyl and conjugated alkenes.[^24^, ^25^] The limited variation makes it challenging to quantify the crosslinking density by FTIR.

We then employed dynamic light scattering (DLS) to analyze the size distributions of the NPs. Following crosslinking for 2–12 h, the average diameter experienced a slight increase from 41 to 46–53 nm, while maintaining a similar narrow size distribution (Figure 2d). The increase in size can be attributed to the crosslinking process, which induced the formation of branched structures and thus making it more difficult for the molecular chains to pack closely in space. In general, the crosslinked fatty acid chains had reduced ability to pack tightly and orderly compared to the non‐crosslinked ones, resulting in a more disordered arrangement and consequently an enlarged particle size.^[26]^ As shown by the differential scanning calorimetry (DSC) curves in Figure S1b, the crosslinked NPs had a melting point (m.p.) at 59.3 °C, which falls slightly below that of stearic acid (m.p.=69.6 °C) but surpasses that of conjugated linoleic acid (m.p.=‐16.0 °C) and lauric acid (m.p.=44.0 °C).^[27]^ As the duration of crosslinking was extended from 2 to 12 h, the m.p. of the crosslinked NPs underwent a gradual elevation, reaching 62.7, 64.2, and 65.5 °C before being stabilized at 65.9 °C. This increase in m.p. can be primarily attributed to the introduction of new covalent bonds as a result of crosslinking, which initiates the formation of a dense network within the particle for enhanced thermal stability. Altogether, it can be concluded that the polymerizable CLAs can be employed to modulate the crosslinking density of NPs made of natural fatty acids. It is anticipated that the formation of a robust covalent network within the NPs will not only enhance their structural stability but also contribute to the modulation of their degradation and release profiles, leading to prolonged and controllable release kinetics.

### Sustained release from the crosslinked nanoscale carriers

The physiological stability of the NPs made of natural fatty acids depends on various factors, including composition, size, surface properties, and the environment they encounter.^[28]^ In many cases, their stability can be compromised by factors such as *i*) potentially enzymatic degradation caused by lipases present in the gastrointestinal tract, which breaks down the NPs for subsequent release of their payloads before reaching the target site and *ii*) in vivo structural failure due to complex pH variations from acidic in the stomach to neutral or slightly alkaline in the intestines.^[29]^ Endowing the NPs with a specific crosslinking density can enhance their physiological stability, reducing susceptibility to degradation in a biological environment.

**Figure 3:**
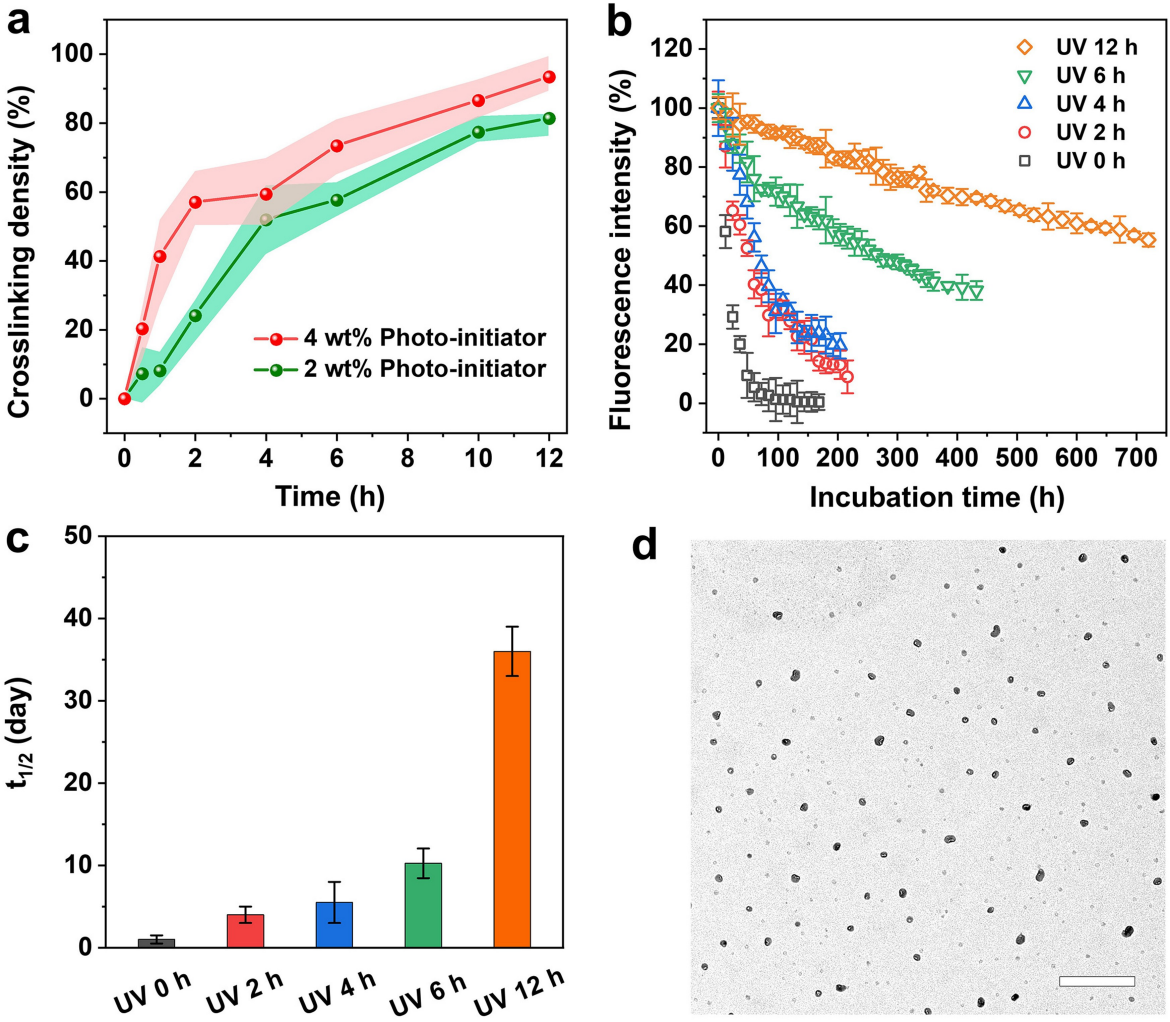
Sustained release from the nanoscale carriers with different crosslinking densities. (a) The crosslinking density of the NPs as a function of UV irradiation time in the presence of 2 and 4 wt % of photo‐initiator, respectively. (b) Fluorescence decay profiles of the NPs loaded with 1‐pyrenedecanoic acid (*ca*. 5 wt %), together with different crosslinking densities (i.e., under UV irradiation for 0, 2, 4, 6, and 12 h), when incubated in PBS containing 10 % FBS. (c) Plots of the degradation half‐life in terms of fluorescence intensity by fitting the curves in (b) to a pseudo‐first order kinetics. (d) TEM image of the NPs with a crosslinking density of 80 % after incubation in the body fluid mimic (PBS containing 10 % FBS) for two weeks. Scale bar: 500 nm.

We varied the UV irradiation time and initiator concentration to elucidate how these factors affect the crosslinking density, a critical determinant that can be defined as the percent of change in absorbances (1‐A_t_/A_0_) at λ=232 nm due to crosslinking (Figure S2). As shown in Figure 3a, the percent increased from 24±2 % to 57±1 % within the initial 2 h of UV exposure, when the amount of initiator was increased from 2 to 4 wt %. This result underscores the inherent kinetics of radical polymerization, such as rapid initiation and subsequent chain propagation. The increase in photo‐initiator tends to generate more primary free radicals, leading to quicker formation of additional crosslinks among polymer chains. Further extending the crosslinking time to 4–12 h resulted in NPs with higher degrees of crosslinking up to 60–95 %. Although 2 or 4 wt % of photo‐initiator was sufficient to initiate the photo‐crosslinking process, a long exposure was required to increase the crosslinking density due to the low power of the light source. This understanding is vital for optimizing the experimental conditions and tailoring the properties of the NPs made of unsaturated fatty acids to meet the requirements of a specific application.

Next, we captured a series of fluorescence micrographs from the NPs loaded with 1‐pyrenedecanoic acid (Figure S3) every 12 or 24 h when they were incubated in PBS (containing 10 % FBS) until the fluorescence from 1‐pyrenedecanoic acid disappeared. The sample was prepared with an encapsulation efficiency (EE) of 11.2±1.8 % and a loading content (LC) of 1.8±0.2 % (Table S1). As documented in the literature,^[30]^ 1‐pyrenedecanoic acid emits strong fluorescence only in a hydrophobic microenvironment. Upon release from the hydrophobic NPs into an aqueous medium, the fluorescence intensity decreases significantly as a result of quenching. The fluorescence intensity was calculated using Image‐J (Figure S4) and then plotted as a function of time to derive the degradation half‐life (Figure 3b). The degradation rate of the NPs was significantly reduced upon exposure to UV light for 2–12 h. In contrast, the control group comprising NPs without crosslinking exhibited rapid degradation, losing 90 % of the fluorescence intensity within two days. When the NPs were crosslinked for 2 h, only 40 % loss in fluorescence intensity was observed during the first three days. Notably, the crosslinked NPs maintained 10 % fluorescence intensity even after extending the incubation time to 10 days. Moreover, when increasing the crosslinking density to 59, 73, and 86 %, the NPs retained 13.9±3.3, 54.1±4.2, and 81.6±3.8 % of fluorescence intensity, respectively, over an incubation period of 10 days.

To further quantify the impact of crosslinking density on the degradation rate of the NPs, we also evaluated the in vitro degradation half‐life by fitting the fluorescence decay profiles in Figure 3b. This metric represents the time required for the fluorescence intensity to diminish to half of its initial value, serving as a gauge of the stability and fluorescence longevity of the NPs. As shown in Figure 3c, the NPs crosslinked for 2 h exhibited a prolonged degradation half‐life (t_1/2_=4.2 days) compared to the non‐crosslinked counterpart (t_1/2_=1.3 day). When the crosslinking time was increased to 4, 6, and 12 h, the corresponding half‐life further increased to 5.5, 10.3, and 36 days, respectively, indicating a progressive enhancement in stability due to more extended crosslinking. This trend suggests that one can increase the crosslinking time to enhance the stability and longevity of the NPs, making them manageable in various applications where prolonged structural integrity and controllable degradation are desired. To elucidate the morphological changes during degradation, we extracted the NPs from the body fluid mimic after a period of 2‐week incubation and analyzed them using TEM. As shown in Figures 3d and S5, the crosslinked NPs exhibited remarkable stability during the 2‐week incubation, whereas the non‐crosslinked NPs underwent fragmentation, resulting in the formation of smaller particles less than 10 nm in diameter.

### Enhanced stability of the crosslinked nanocarriers inside cells

**Figure 4:**
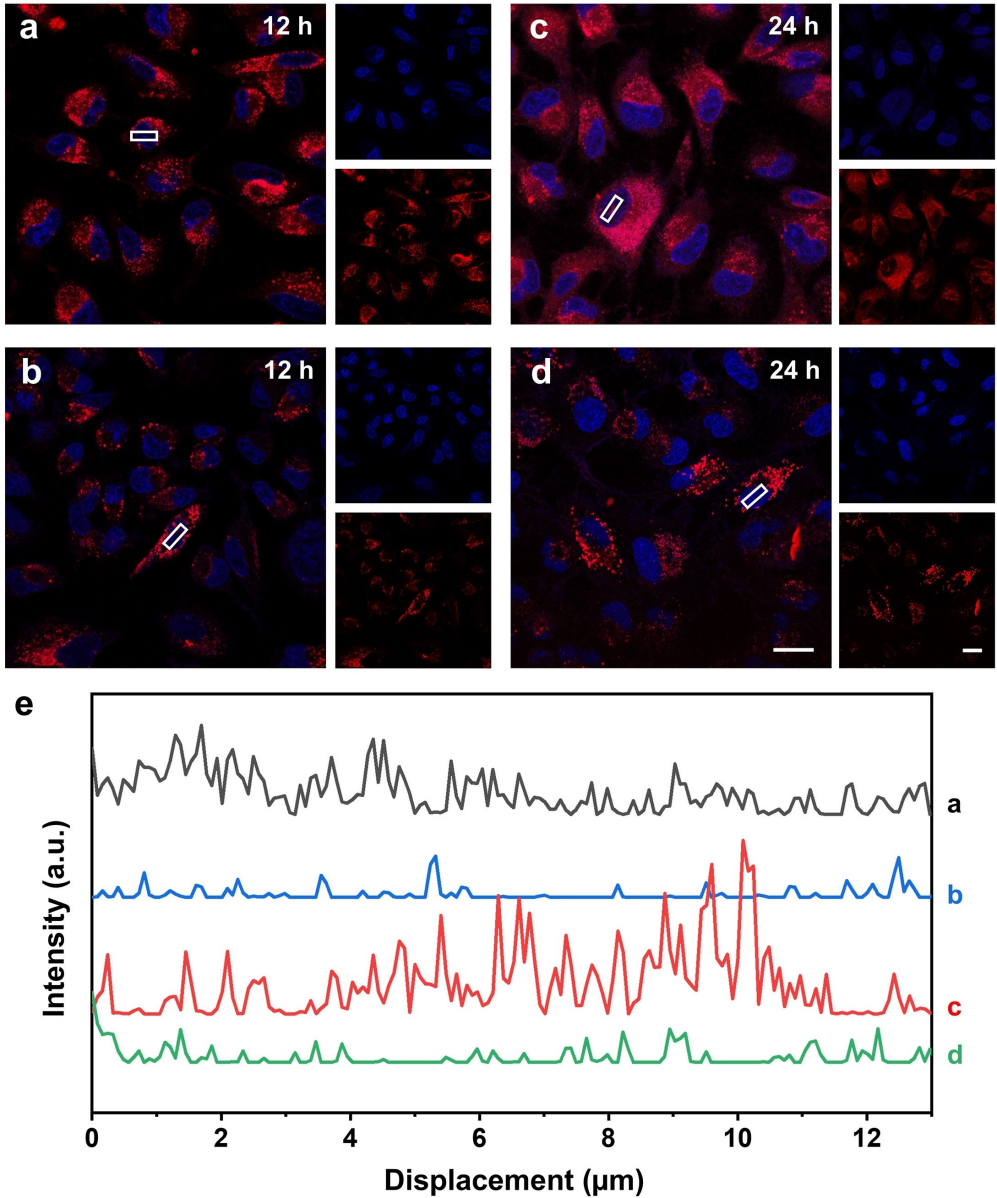
Enhanced stability of the crosslinked nanocarriers inside cells. (a–d) Fluorescence images showing the distributions of the (a, c) non‐crosslinked and (b, d) crosslinked DOX‐loaded NPs (*ca*. 0.20 mg/mL) inside A549 cells after incubation for 12 and 24 h, respectively. The cell nucleus was stained with Hoechst 33342 (blue) while DOX gave a red color. Scale bars: 20 μm. (e) Quantitative analysis of DOX fluorescence intensity over the nucleus for the two groups treated with non‐crosslinked and crosslinked NPs for 12 and 24 h, respectively. The intensities were measured along the boxes marked on the fluorescence images. The curves were vertically shifted for a clear display.

Unstable NPs are prone to premature burst release of their payloads inside cells, resulting in the loss of advantages associated with controlled release and thus ineffective therapy or potential toxicity due to overdosing.^[31]^ To this end, we further investigated the intracellular stability of the crosslinked NPs loaded with DOX in A549 cells by tracking the fluorescence intensity of DOX molecules (EE: 8.2±0.8 % and LC: 1.4±0.2 %, Table S1). Both types of DOX‐loaded NPs emitted red fluorescence, allowing us to monitor their cellular uptake and intracellular distribution (Figure S6). After incubation for 12 h, both non‐crosslinked and crosslinked NPs accumulated near the perinuclear region of the cell, resulting in concentrated fluorescence emission surrounding the nucleus (Figure 4a, b). However, significant differences emerged after 24 h post‐incubation (Figure 4c, d). The non‐crosslinked NPs exhibited a diffuse fluorescence pattern, indicating the release of DOX from the NPs, likely due to their instability in the lysosomal environment.^[32]^ In contrast, the crosslinked NPs retained a stable and localized fluorescence near the nucleus, suggesting enhanced intracellular stability and retarded release of DOX. This stability likely stems from the crosslinked network that makes the NPs resistant to degradation by the acids and enzymes from the lysosomes. According to the quantitative analysis in Figure 4e, at 24 h, the average red fluorescence intensity in the nuclear region of the group treated with non‐crosslinked NPs was four times higher than that of the group treated with crosslinked NPs. This trend indicates that most of the DOX remained in the crosslinked NPs over an extended period of 24 h. Taken together, the enhanced stability observed for the crosslinked NPs confirms that crosslinking of fatty acids offers a promising strategy to prolong drug retention inside endolysosomal compartments, enhancing the efficacy and precision of drug delivery, such as preventing cancer recurrence after surgery.

**Figure 5:**
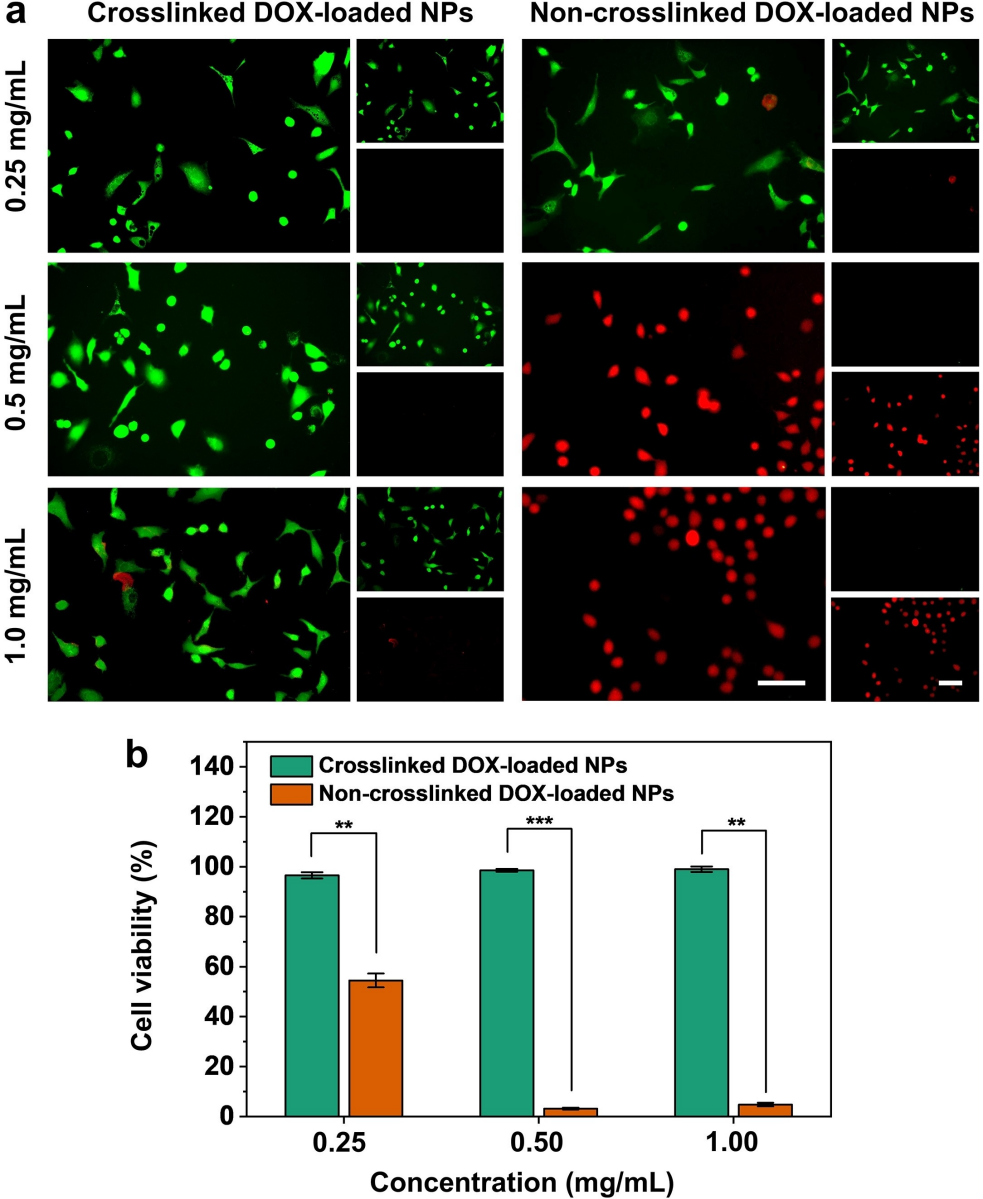
Cytotoxicity of the crosslinked and non‐crosslinked DOX‐loaded NPs. (a) Fluorescence images of A549 cells cultured with NPs at concentrations of 0.25, 0.5, and 1.0 mg/mL for 1 day. Scale bars: 100 μm. (b) Cell viability of A549 cells incubated with the NPs at different concentrations. Error bars represent standard deviation for *n*=5. **P<0.01 and ***P<0.005, determined by T‐test.

We hypothesized that the crosslinked NPs would exhibit relatively low cytotoxicity even when loaded with DOX due to the retarded release profile. To validate this hypothesis, we measured the release profiles of DOX loaded in the non‐crosslinked and crosslinked NPs, respectively, when suspended in water. As shown in Figure S7, the amount of the DOX released from the crosslinked NPs was less than 20 % within 10 days, significantly lower than the approximately 95 % release observed for the non‐crosslinked counterpart. Next, we evaluated the cytotoxicity of the DOX‐loaded NPs using live/dead fluorescence imaging and cell viability assay. As shown in Figure 5a, the non‐crosslinked NPs exhibited a clear dose‐dependent cytotoxicity against A549 cells at concentrations of 0.25, 0.5, and 1.0 mg/mL. At concentrations of 0.5 and 1.0 mg/mL, the cell viability dropped from 95 % to less than 10 %. In contrast, even at the highest tested concentration (1.0 mg/mL), the crosslinked NPs showed no obvious cytotoxicity on A549 cells. This trend suggests that the crosslinking strategy stabilizes the NPs, potentially reducing the release rate of the payload and thus minimizing its cytotoxicity. The quantitative effect of the DOX‐loaded NPs on A549 cell viability was also validated using the MTT assay. This assay measures cell metabolic activity by exploiting the ability of viable cells, which contain NAD(P)H‐dependent oxidoreductase enzymes, to reduce MTT to formazan. At a concentration of 0.25 mg/mL, the non‐crosslinked NPs induced a half‐maximal absorbance in the MTT assay, indicating that approximately 45 % of the A549 cell population was killed (Figure 5b). As the concentration of the non‐crosslinked NPs was increased, cytotoxicity also increased, in agreement with the results obtained from the live/dead staining method. However, the cell viability remained at 100 % across all tested concentrations of the crosslinked NPs, confirming their enhanced stability during incubation and cellular internalization. This difference in cytotoxicity between the non‐crosslinked and crosslinked samples suggests that crosslinking could be a crucial strategy in designing safer and better controlled drug delivery systems. While non‐crosslinked NPs may be useful for applications requiring rapid and potent cell killing,[^33^, ^34^] the crosslinked ones would offer a safer alternative for chronic treatments, where prolonged and controlled drug release with minimal side effects is desired.^[35]^

### Neurite outgrowth in the presence of a neurotrophin released from microscale carriers

For the repair and regeneration of peripheral nerves, a promising strategy is the delivery of neurotrophins that can influence the local function of cells within and surrounding the injured site over an extended period, ranging from several weeks to months.[^36^, ^37^, ^38^] By utilizing the crosslinking strategy for microparticles made of unsaturated fatty acids, we can further modulate the release profile of the neurotrophins and thereby influence the extension of neurites. To validate this hypothesis, we used a coaxial electrospray method (Figure S8) to prepare the core–shell microparticles made of unsaturated fatty acids, together with human recombinant neurotrophin‐3 (NT‐3) in the core, and then crosslinked the microparticles to a certain extent through the use of UV irradiation. Figure S9 shows fluorescence micrographs of the microparticles. To visualize the core–shell structure, indocyanine green was incorporated into the core, while FITC‐BSA was incorporated into the shell for easy fluorescence analysis. The co‐localization of green and red colors and the appearance of a red edge around each particle confirms a core–shell structure, demonstrating that the payloads were successfully loaded in the core. We then incubated the non‐crosslinked or crosslinked microparticles with neurobasal plus medium for seven days and obtained supernatants containing different amounts of released NT‐3. ELISA tests based on the standard curve in Figure S10 indicate that the concentrations of released NT‐3 were 9.44 for the non‐crosslinked and 2.24 ng/mL for the crosslinked microparticles, respectively, confirming the slower release of the crosslinked microparticles.

**Figure 6:**
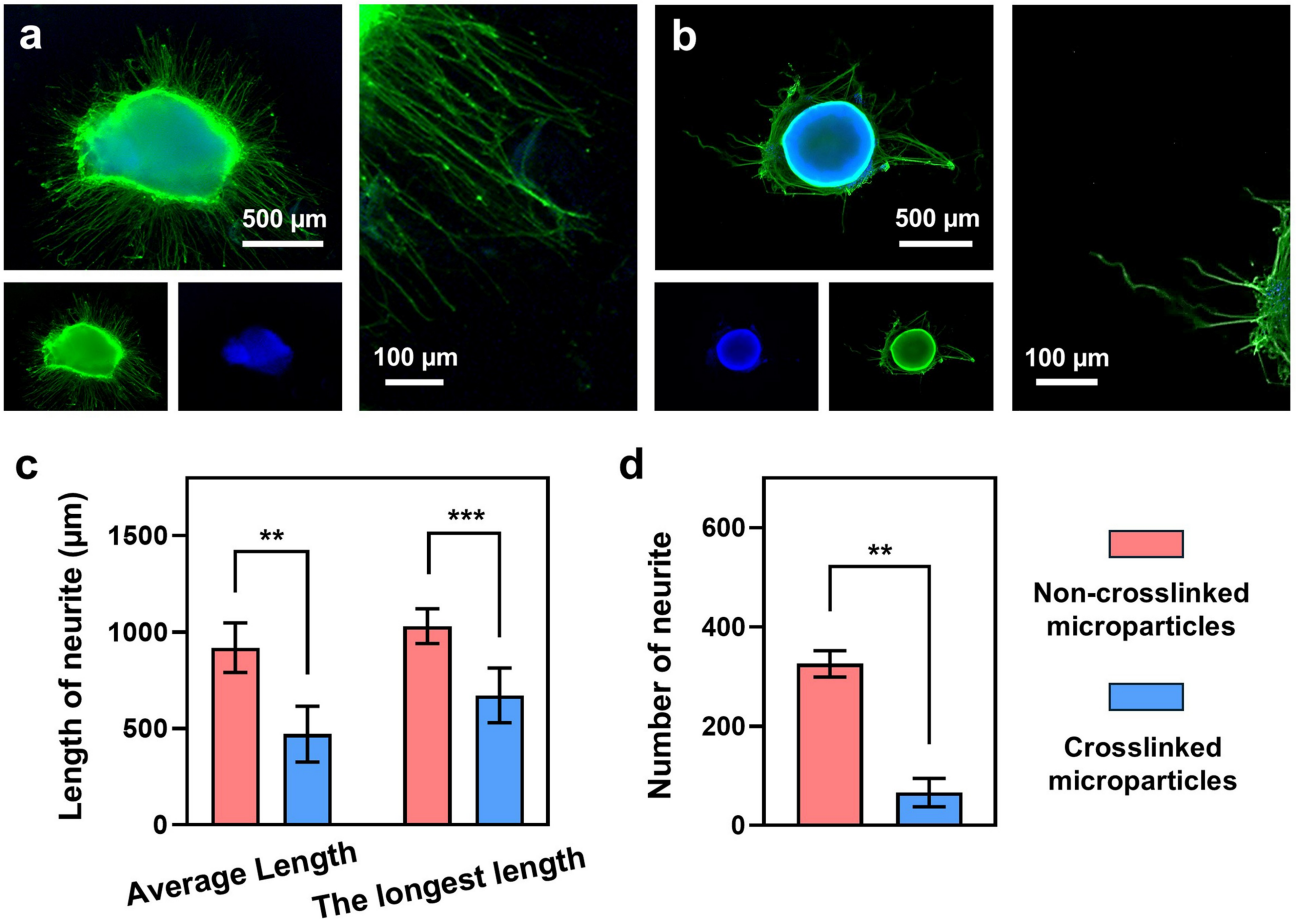
Neurite outgrowth from DRG in the presence of NT‐3 released from the microparticles based on fatty acids. Fluorescence images showing neurite fields extending from DRG when cultured in a laminin‐coated 12‐well plate for 7 days, with the treatment of a mixture containing 900 μL of a neurobasal plus medium (supplemented with 10 % FBS, 1 % N‐2 supplement, and 1 % ABAM) and 100 μL of the NT‐3 solution released from the microparticles made of (a) non‐crosslinked and (b) crosslinked fatty acids. The neurites were stained with Tuj1 marker (green) while the nuclei were identified by DAPI labeling (blue). The right panels are the enlarged versions. (c) Average neurite length, maximum neurite length and (d) number of extended neurites from DRG when cultured with the NT‐3 released from non‐crosslinked and crosslinked microparticles, respectively (*n*=6). ** p<0.01 and *** *P*<0.005.

Next, we investigated the influence of the released NT‐3 on neurite outgrowth from chick dorsal root ganglion (DRG). For each group, embryonic chick DRG bodies were seeded in the center of a laminin‐coated 12‐well plate. During culture, we replaced the medium with a mixture of 900 μL of neurobasal plus medium (supplemented with 10 % FBS, 1 % N‐2 supplement, and 1 % antibiotic antimycotic (ABAM)) and 100 μL of the released NT‐3 solution in each well every 2 days. Figure 6a, b shows fluorescence micrographs of neurites projecting from the DRG bodies after incubation for 7 days. The right panels show images at a higher magnification to more clearly reveal the morphology of the neurites. As indicated by the data in Figures 6c, the average and maximum lengths of the neurites treated with the solution released from the non‐crosslinked microparticles were 919±128 and 1030±90 μm, respectively. These lengths were significantly greater than those observed in the crosslinked group, which gave an average length of 471±145 μm (*P*<0.01) and the longest length of 671±141 μm (*P*<0.005), respectively. Furthermore, the total number of neurites for the group treated with the solution released from non‐crosslinked microparticles was 325, which was nearly 3.9 times greater than the number observed in the crosslinked group (*P*<0.01, Figure 6d). These observations confirm that crosslinking was able to stabilize the microparticles, resulting in less degradation of fatty acids and more sustained release of NT‐3, which in turn slowed down the rate of neurite outgrowth. When the DRG bodies were cultured without the addition of the NT‐3 solution released from the microparticles (Figure S11), no significant neurite extension was observed, highlighting the crucial role of NT‐3 and potentially dissolved fatty acids in stimulating neurite outgrowth. Taken together, we have successfully applied the crosslinking strategy to microparticles made of fatty acids, enabling controlled neurite extension by modulating the release profile of neurotrophins. This approach can optimize neurite outgrowth and support the overall peripheral nerve regeneration process by maintaining a stable and supportive environment for the neurons.

## Conclusions

In summary, we have developed a simple strategy to finely tune the crosslinking density of carriers made of natural fatty acids. The branched structure formed during crosslinking allows the carriers to maintain a low crystallinity upon solidification, significantly improving drug loading efficiency. Unlike the previously reported carriers based on non‐crosslinked fatty acids, the current system exhibits controlled release kinetics tailored to different applications. In vitro degradation analysis suggests that the crosslinked nanocarriers had remarkable stability even after 2‐week incubation in a body fluid mimic. In a proof‐of‐concept experiment, despite being loaded with DOX, the crosslinked nanocarriers show no discernible cytotoxic effects on A549 cells up to 24 h, highlighting their enhanced intracellular stability. We further extend the crosslinking strategy to microparticles featuring a core–shell structure and comprising NT‐3 and unsaturated fatty acids. The observed slower outgrowth of neurites from DRG confirmed that the crosslinked network effectively retards the release of NT‐3. This work not only establishes a fundamental approach for stabilizing carrier materials based on fatty acids but also unveils new avenues for their applications in drug delivery and related areas, where prolonged drug release with minimal side effects is desired.

## Conflict of Interests

The authors declare no conflict of interest.

1

## Supporting information

As a service to our authors and readers, this journal provides supporting information supplied by the authors. Such materials are peer reviewed and may be re‐organized for online delivery, but are not copy‐edited or typeset. Technical support issues arising from supporting information (other than missing files) should be addressed to the authors.

Supporting Information

## Data Availability

The data that support the findings of this study are available from the corresponding author upon reasonable request.
