# Supplementary material for: Nanocarriers Made of Natural Fatty Acids: Modulation of Their Release Profiles through Photo‐Crosslinking
Source: Angew Chem Int Ed Engl. 2024 Dec 4;64(3):e202415671. doi: 10.1002/anie.202415671 (PMC11735881; doi:10.1002/anie.202415671)
Supplement: Supplementary file 1 — Supporting Information [file ANIE-64-e202415671-s001.pdf]

Angewandte Chemie

Eine Zeitschrift der Gesellschaft Deutscher Chemiker

Supporting Information

Nanocarriers Made of Natural Fatty Acids: Modulation of Their Release Profiles through Photo-Crosslinking

*D. Zhang, Y. Meng, M. Hao, Y. Xia**

Support Information

Nanocarriers Made of Natural Fatty Acids: Modulation of Their Release Profiles through Photo-Crosslinking

Dong Zhang,[a] Yuxuan Meng,[b]] Min Hao,[a] and Younan Xia*, [a,b]

[a] Prof. Dr. Y. Xia, Dr. D. Zhang, Dr. M. Hao
The Wallace H. Coulter Department of Biomedical Engineering, Georgia Institute of Technology and Emory University, Atlanta, GA 30332 (USA)
E-mail: younan.xia@bme.gatech.edu

[b] Prof. Y. Xia, Y. Meng
School of Chemistry and Biochemistry, Georgia Institute of Technology, Atlanta, GA 30332 (USA)

Experimental section

Chemicals and materials. Lauric acid (LA, >97%), stearic acid (SA, >95%), conjugated linoleic acids (CLAs, a mixture of *cis*- and *trans*-9,11- and -10,12-octadecadienoic acids, >99%), 2,2-dimethoxy-2-phenylacetophenone (99%), doxorubicin hydrochloride (DOX, >99%), and anhydrous ethanol (>99.5%) were all purchased from Sigma-Aldrich. 1-pyrenedecanoic acid (99%) was obtained from Abcam. Fetal bovine serum (FBS), RPMI 1640 medium, neurobasal plus medium, N-2 supplement, antibiotic antimycotic (ABAM), and phosphate buffered saline (PBS) were ordered from Thermo Fisher Scientific. 1,2-distearoyl-*sn*-*glycero*-3-phosphoethanolamine-*N*-[methoxy (polyethylene glycol)-5000] ($DSPE\text{-}PEG_{5k}$) was obtained from Laysan Bio.

Preparation of the NPs. The NPs were prepared by contacting the fatty acids in ethanol with an anti-solvent (water) through hydrodynamic flow focusing in a homemade fluidic device.[1] The focused phase was prepared by dissolving LA, SA, and CLAs in ethanol to form a solution with a total fatty acid concentration of 6.0 mg/mL, along with 2,2-dimethoxy-2-phenylacetophenone (2–4 wt% of CLAs) as an initiator. The mass ratio between LA to SA was maintained at 1:5, while the CLAs content was set to 5 or 10 wt% relative to the total amount of LA and SA. The focusing phase was formulated by dissolving $DSPE\text{-}PEG_{5k}$ in water to form a solution with a concentration of 0.5 mg/mL. The lipid solution and fatty acid solution were introduced using two syringe pumps (KD 100, KD scientific), each operating at an independently controlled flow rate. The volumetric flow rate of the focusing phase was set to 700 μL/min, while the ratio between the flow rates of the focusing and focused phases was controlled at 50:1. For the measurement of degradation profile, 1-pyrenedecanoic acid was incorporated into the NPs as a fluorescent probe. Typically, the 1-pyrenedecanoic acid was dissolved in ethanol (1.0 mg/mL) and then mixed with the focused phase to achieve the desired concentration. Using a similar strategy, NPs loaded with DOX (1.0 mg/mL) were also synthesized. The as-prepared NPs underwent centrifugation at 14,000 rpm (6 min) and were then washed with water three times to remove the ethanol and un-encapsulated molecules. The NPs were resuspended in water for further use.

Photo-crosslinking of the NPs. To enhance the structural integrity and stability of the NPs, the sample was subjected to crosslinking by UV irradiation (365 nm, 4 W) in a glove box filled with N_2 for different periods of time (*i.e.*, 2, 4, 6, 10, and 12 h). We used glass vials (Wheaton Scintillation, Type I borosilicate glass) purchased from VWR to allow the UV light to pass through the wall.

Characterizations. The NPs were analyzed using a transmission electron microscope (TEM, Hitachi 7700). The extent of crosslinking in the NPs was determined by following the UV-vis absorption peak of the CLAs. To further measure the conjugated double bonds in the NPs, Fourier-transform infrared (FTIR) spectra were recorded in the spectral region from 400–4000 cm^{-1}. The particle size distribution was assessed using dynamic light scattering (DLS, Zetasizer Nano ZS, Malvern) at 25 °C. Differential scanning calorimetry (DSC, TA-250, TA Instruments) was employed to test the thermal properties of the NPs before and after photo-crosslinking. Samples weighed between 1–10 mg were first equilibrated at 25 °C and subsequently subjected to heating at a rate of 10 °C per minute under a N_2 atmosphere and a purge flow of 50 mL/min. Payload concentrations were determined through UV-vis spectral analysis (Cary 60, Agilent Technologies) of the NPs dissolved in ethanol, followed by quantification using the corresponding calibration curves (488 nm for DOX, 275 or 340 nm for 1-pyrenedecanoic acid). The encapsulation efficiency (EE) and loading content (LC) were determined using Equations (1) and (2), respectively:

$$EE = \frac{W_0}{W_{drug}} * 100\% \qquad (1)$$

$$LC = \frac{W_0}{W_{NP}} * 100\% \qquad (2)$$

where W_0, W_{drug}, and W_{NP} represent the weight of the drug in NPs, the weight of the drug added, and the weight of the NPs, respectively.

Determination of the in vitro release profile. To investigate the release kinetics of the NPs, we incubated an aqueous suspension (0.5 mg/mL) of NPs pre-loaded with 1-pyrenedecanoic acid in a PBS solution containing 10% FBS. In a typical protocol, 200 □L of an aqueous suspension of the NPs (0.5 mg/mL) was placed in each well of a 12-well plate, followed by drying. Afterwards, 1 mL of the PBS (containing 10% FBS) was added, and a series of fluorescence micrographs were acquired from the NPs deposited at the bottom using an inverted microscope (DMI6000, Leica) at a 12- or 24-h interval for up to 5 weeks. Subsequently, the fluorescence intensity was plotted as a function of time to determine the degradation half-life.

Cell culture. We cultured A549 human non-small cell lung cancer cells in the Roswell Park Memorial Institute 1640 (RPMI 1640) medium supplemented with 10% FBS and 1% antibiotics (penicillin and streptomycin). The cells were incubated at 37 °C in a humidified atmosphere with 5% CO_2 and the medium was refreshed every two days to maintain optimal conditions for cell proliferation.

Fluorescence microscopy characterization. A549 cells (100 μL) were seeded in a 96-well plate at a density of 1.0 × 10^4 cells per well and cultured for 24 h. Afterwards, 20 μL of the suspension of the non-crosslinked or crosslinked NPs (0.2 mg/mL) were added, followed by incubation at 37 °C for 12 and 24 h, respectively. After washing with PBS once, the cells were stained with Hoechst 33342 (10 μg/mL) in RPMI 1640 at 37 °C for 10 min. After washing with PBS three times, fresh culture medium was supplemented prior to fluorescence microscopy imaging. The fluorescence micrographs were captured using a confocal laser scanning microscope (Zeiss LSM 900) and further analyzed with Image-J software.

Cell viability assay. A549 cells were seeded in a 96-well plate at a density of 1.0–1.5 × 10^4 cells per well and cultured overnight. 20 μL of an aqueous suspension of the crosslinked or non-crosslinked NPs with varying concentrations (0.25, 0.5, and 1.0 mg/mL, n = 5 for each group) were added, followed by incubation at 37 °C. After culture for one day, 100 μL of MTT solution (0.5 μg/mL in culture medium) was added into each well. The plates were further incubated at 37 °C for 4 h. Subsequently, the supernatant in each well was removed and replaced with 100 μL of DMSO to dissolve the formazan crystals. The absorbance of the resulting solution was measured at 490 nm using a plate reader (Infinite 200, TECAN). For live/dead fluorescence imaging, the cells were stained with a live/dead kit in PBS at room temperature for 20 min, followed by washing with PBS three times. Fluorescence micrographs were acquired using an optical microscope (DMI6000, Leica) and analyzed with Image-J software.

Fabrication of microparticles made of fatty acids and loaded with Human Recombinant Neurotrophin-3 (NT-3). To demonstrate the generality of the crosslinking strategy, we further prepared microparticles made of fatty acids and loaded with NT-3 using a coaxial electrospray method (Figure S7).[2] The resultant microparticles had a core-shell structure. Briefly, NT-3 was dissolved in 0.5 wt% aqueous gelatin solution and used as the inner solution. The fatty acids (a mixture of LA, SA, and CLA at a mass ratio of 20:100:1, supplemented with 2 wt% 2,2-dimethoxy-2-phenylacetophenone) were dissolved in an ethanol/dichloromethane mixture (20:80 by vol.) at a total concentration of 10 wt% and utilized as the outer solution. The feeding rates of the outer and inner solutions were controlled at 1.5 and 0.5 mL/h, respectively. A high voltage (DC) of 20 kV was applied to the coaxial spinneret, with a glass slide serving as the collector. The collected microparticles were exposed to UV irradiation (365 nm, 4 W) for 6 h in a glove box filled with N_2. To visualize the core-shell structure, we added indocyanine green and FITC-BSA into the inner

and outer solutions prior electrospray, followed by fluorescence imaging.

Release of NT-3 from the microparticles. The sterile microparticles, both crosslinked and non-crosslinked, were incubated in 1.0 mL of neurobasal plus medium at 37 °C. After seven days, the medium was cooled to 4 °C, and the supernatants containing different amounts of released NT-3 were extracted. Each sample of solution was retrieved and stored at -20 °C before further use. The NT-3 level in each group was measured using an ELISA kit (Thermo Fisher Scientific) according to the manufacturer's protocol, with triplicate samples for each group.

Isolation and culture of dorsal root ganglions (DRG). We harvested DRG from the thoracic region of the spinal column in embryonic white leghorn chicks obtained from a nearby farm. Using precise micro-dissection tools, we carefully isolated DRG from the thoracic spine of day 8 embryos by ensuring minimal damage to the ganglia. The isolated DRG were then placed in a 12-well plate pre-coated with laminin and cultured in a neurobasal plus medium supplemented with 10% FBS, 1% N-2 supplement, and 1% ABAM. We replaced the culture medium in each well with a fresh mixture of 900 µL of neurobasal plus medium (supplemented with 10% FBS, 1% N-2 supplement, and 1% ABAM) and 100 µL of the release solution every 24-48 h. At day 7 of incubation, the neurites were immunostained with an anti-βIII tubulin (Tuj1) primary antibody and an Alexa Fluor 488 secondary antibody, while the cell nuclei were stained with DAPI. Fluorescence micrographs were then captured using a laser confocal scanning microscope (Zeiss LSM 900, Carl Zeiss, Germany). The average and longest lengths of neurites were quantitatively analyzed from the fluorescence microscopy images using ImageJ software. Each group was studied with six DRG bodies.

Note: The rationale for using pre-prepared NT-3 release solution instead of directly adding nanocarriers was as follows. The overall culture period for DRG was 7 days, with the medium being changed 1−2 days. If the carriers (microparticles) were directly added into the culture, they would be inevitably removed along with the old medium during the medium exchange process, leading to their loss and thus compromising the fairness of comparison. In addition, the NT-3 released from the carriers may form local gradients because of the absence of shaking/stirring and thus inadequate diffusion, if the carriers were directly added. Using the release solution instead, the concentration of NT-3 involved in each group remained consistent and uniformly distributed during the culture process, allowing for more accurate and meaningful comparison.

Statistical analysis. All statistical values were averaged from at least triplicate samples and

presented as mean ± standard deviation, with "*n*" indicating the number of samples per group. Comparisons between groups were performed using one-way ANOVA, followed by Student's t-tests for all pairwise comparisons. Differences were considered statistically significant when $P < 0.05$.

References

[1] Q. Chen, C. Zhu, D. Huo, J. Xue, H. Cheng, B. Guan, Y. Xia, *Nanoscale* **2018**, *10*, 22312–22318.

[2] J. Xue, C. Zhu, J. Li, H. Li, Y. Xia, *Adv. Func. Mater*. **2018**, *28*, 1705563.

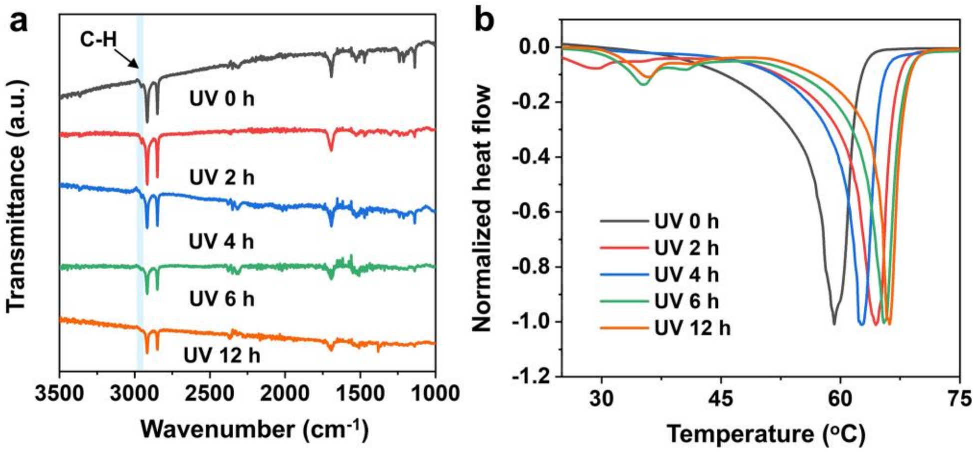

Figure S1. (a) FTIR spectra and (b) DSC curves of the NPs (dry powders) before and after UV irradiation for different periods of time (*i.e.*, 2, 4, 6, and 12 h).

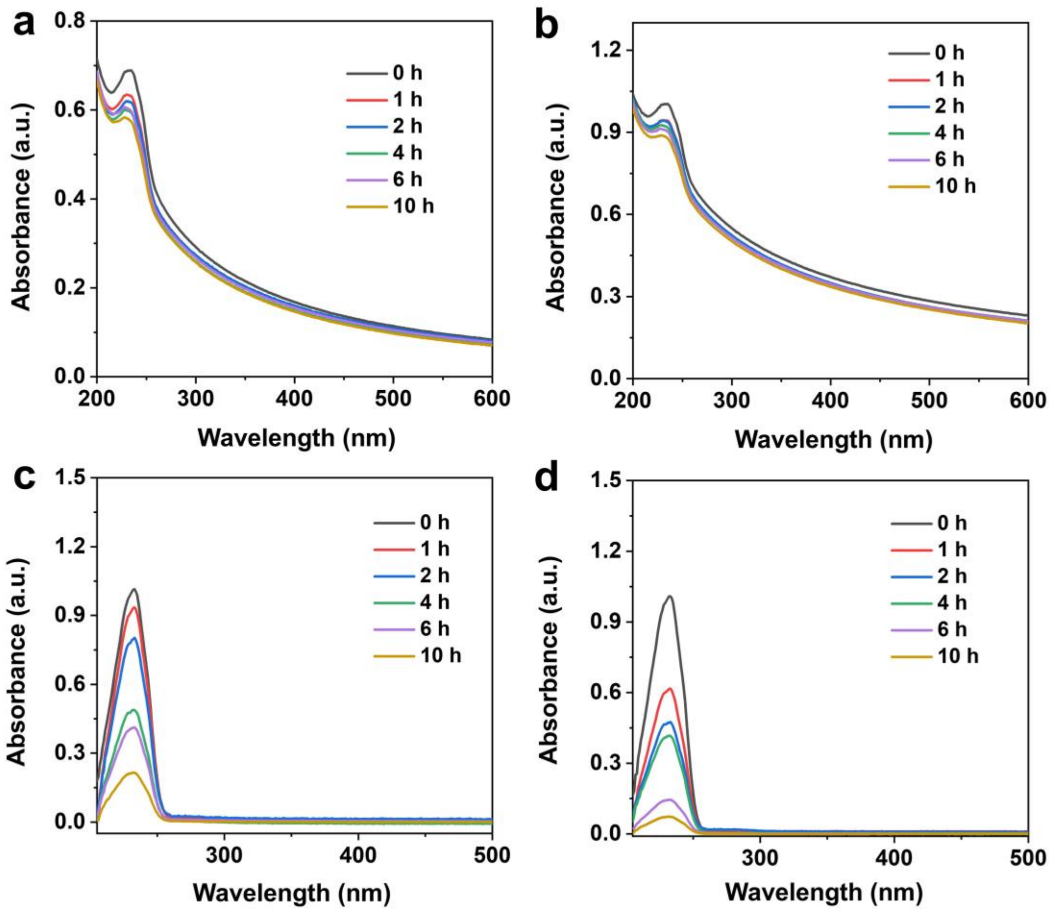

Figure S2. UV-vis absorbance spectra of (a, b) an aqueous suspension of NPs made of fatty acids with varying degrees of crosslinking (*i.e.*, under UV irradiation for 0, 1, 2, 4, 6, and 10 h) and (c, d) a mixture of the corresponding NPs dissolved in ethanol. The NPs were crosslinked in the presence of (a, c) 2 and (b, d) 4 wt% of photo-initiators. For the NP solution in ethanol, the sample was prepared as follows: 10 µL of the aqueous suspension of the non-crosslinked or crosslinked NPs (*ca*. 0.12 mg/mL) was dissolved in 1 mL of ethanol before testing.

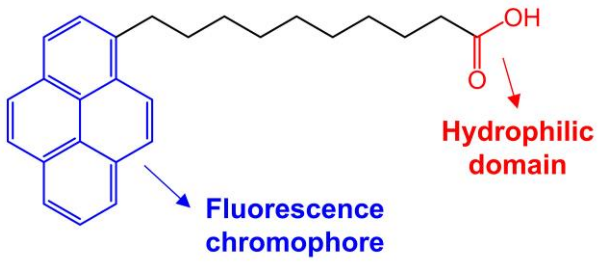

Figure S3. Schematic illustrating the chemical structure of 1-pyrenedecanoic acid.

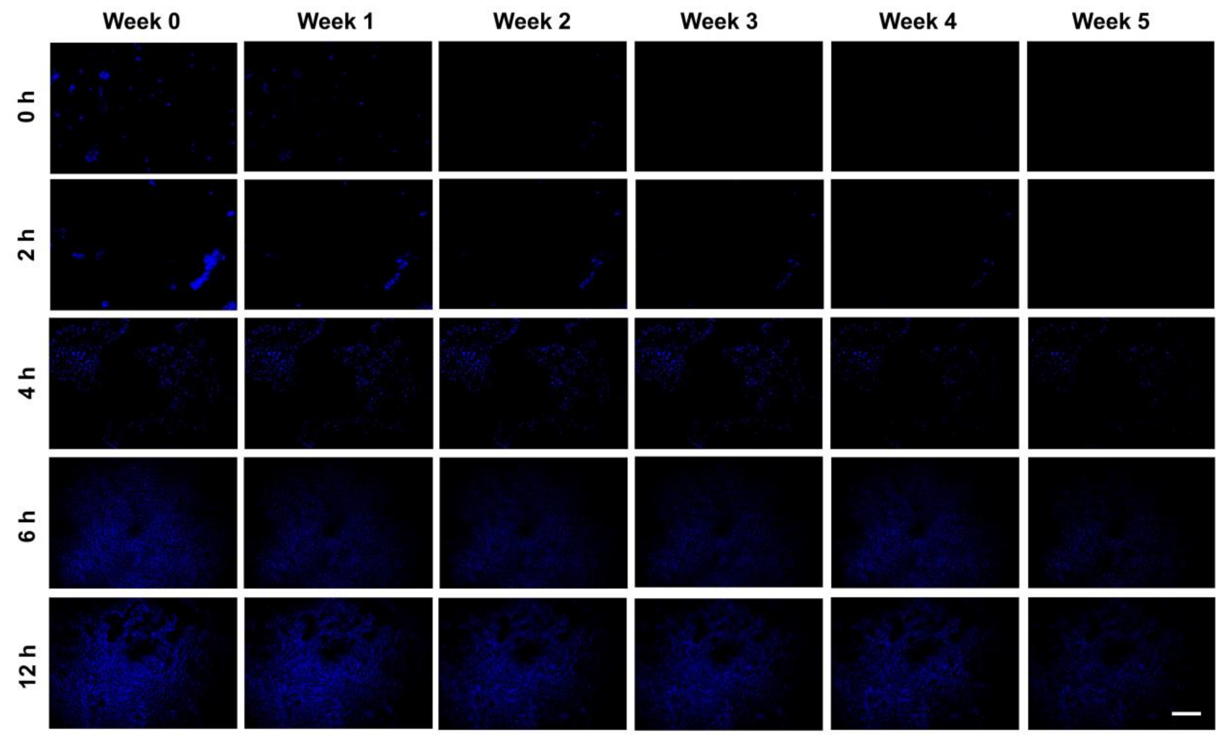

Figure S4. Fluorescence micrographs recorded from the NPs containing 1-pyrenedecanoic acid with varying degrees of crosslinking (*i.e.*, under UV irradiation for 0, 2, 4, 6, and 12 h) after incubation in PBS containing 10% of FBS for five weeks. Scale bars: 100 μm.

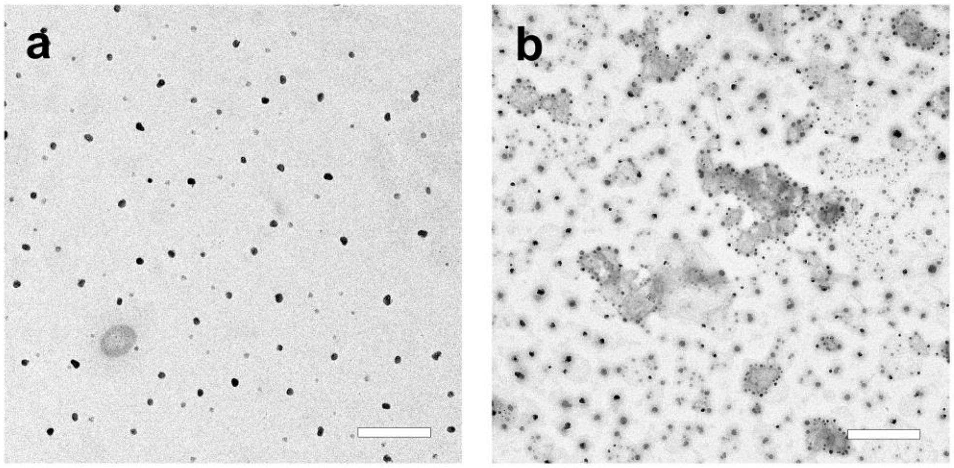

Figure S5. TEM image of the non-crosslinked NPs (a) before and (b) after incubation in PBS containing 10% FBS for two weeks. Scale bar: 500 nm.

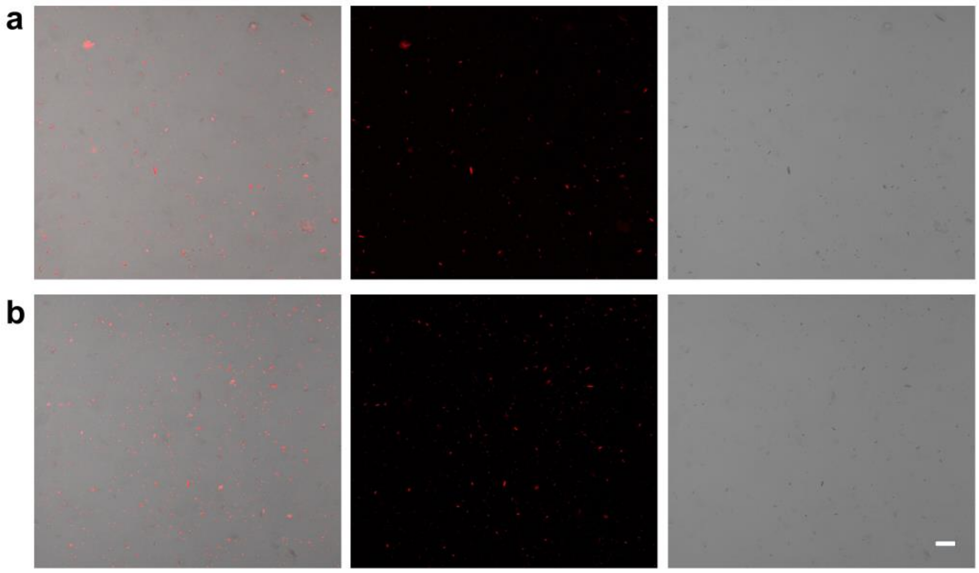

Figure S6. Micrographs (left column) formed by merging the bright-field (grey, right column) and fluorescence (red, middle column) images recorded from (a) non-crosslinked and (b) crosslinked DOX-loaded NPs, respectively. The red fluorescence was emitted from DOX upon excitation. The scale bar is 20 μm and applies to all panels.

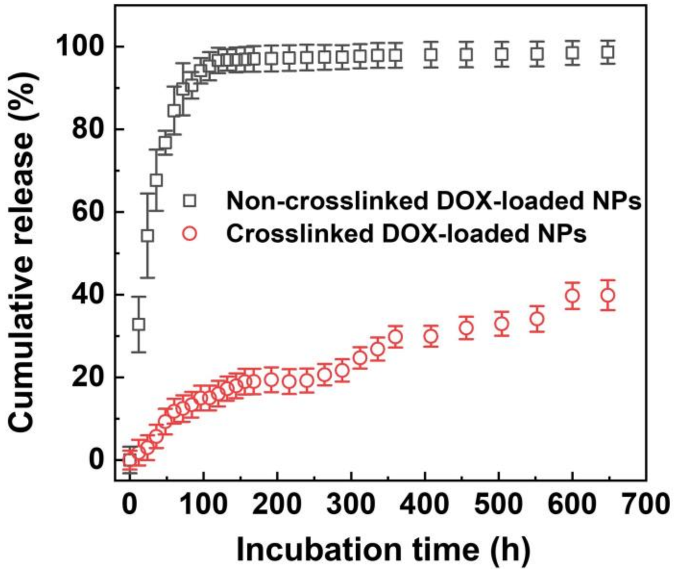

Figure S7. Release profiles for DOX from the non-crosslinked and crosslinked (under UV irradiation for 10 h) NPs (n=3), respectively, in PBS containing 10% FBS. Cumulative DOX release (%) was defined as the ratio between the amount of DOX released at a given time and the theoretical initial amount encapsulated.

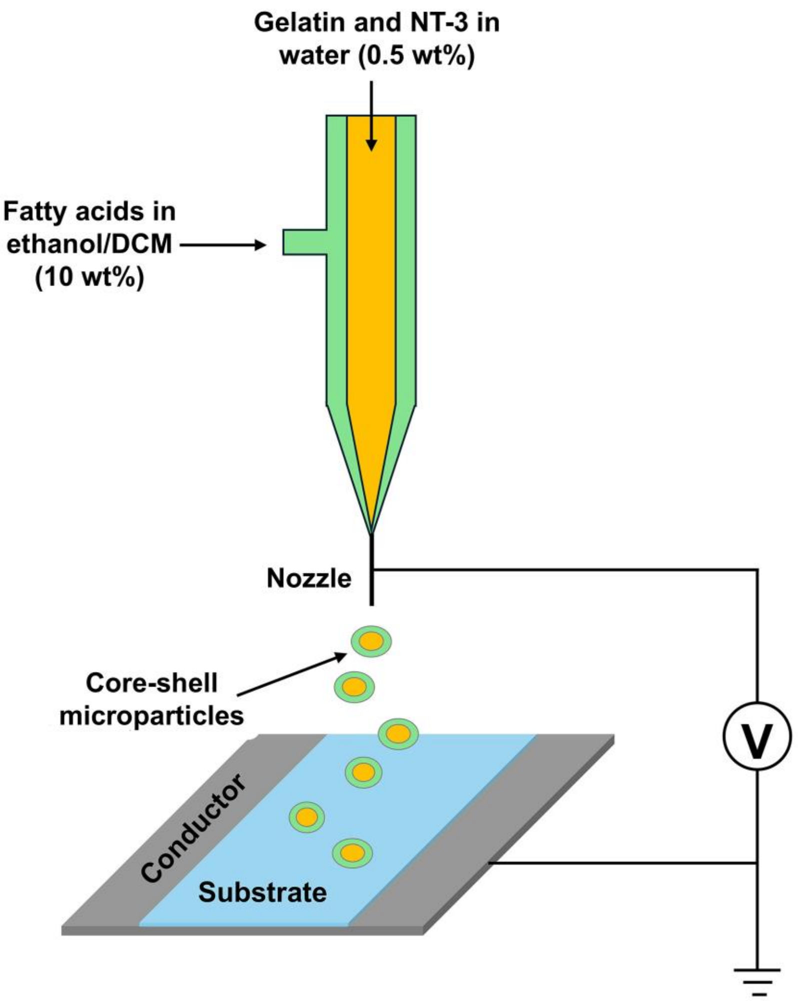

Figure S8. Electrospray set-up for the fabrication of microparticles made of fatty acids and loaded with human recombinant neurotrophin-3 (NT-3).

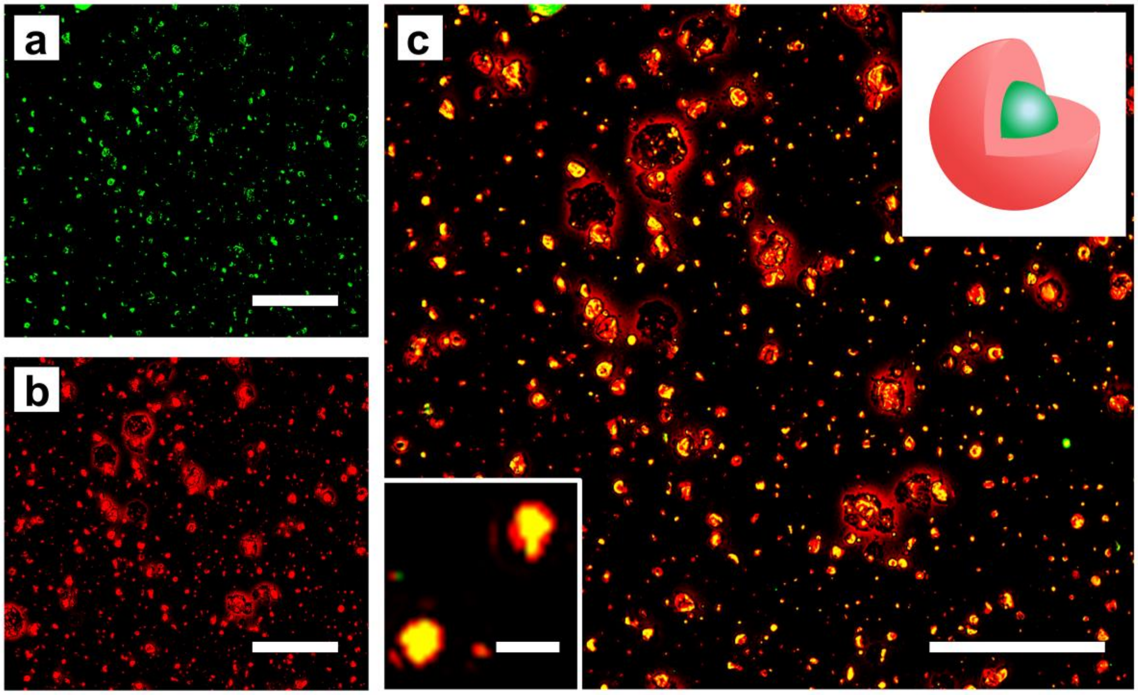

Figure S9. Fluorescence micrographs of gelatin-NT-3@fatty acids microparticles featuring a core-shell structure prepared *via* coaxial electrospray. The core (a) contained ICG (green), whereas FITC-BSA (red) was incorporated in the shell (b). The merged micrograph in (c) confirms the appearance of an orange color in the center of each particle due to a combination of fluorescence colors from ICG and FITC-BSA whereas the edge still showed a red color corresponding to FTIC-BSA. Scale bars in the fluorescence images and the inset in (c) are 100 and 5 µm, respectively.

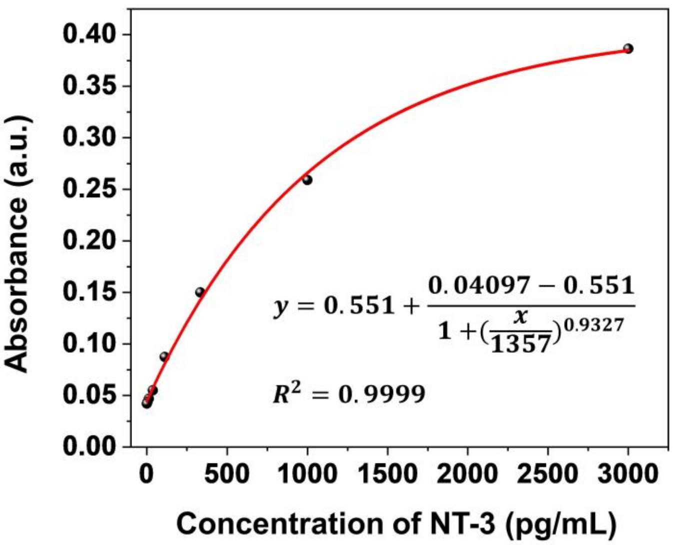

Figure S10. Calibration curve used for determining the concentration of NT-3 through the use of an ELISA kit.

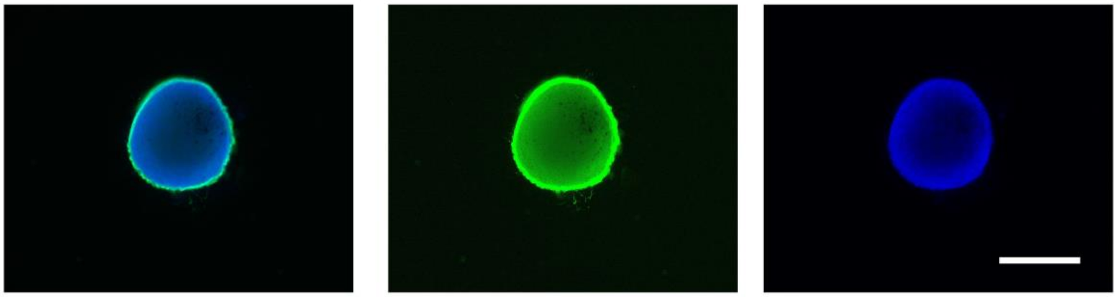

Figure S11. Merged fluorescence image (left) showing neurite fields extending from DRG when culture in a laminin-coated 12-well plate for 7 days, with the treatment of 1000 µL of a neurobasal plus medium (supplemented with 10% FBS, 1% N-2 supplement, and 1% ABAM). The neurites were stained with Tuj1 marker (green, middle panel) while the nuclei were identified by DAPI labeling (blue, right panel). Scale bars: 500 nm.

Table S1. Summary of the encapsulation efficiency (EE) and loading capacity (LC) of 1-pyrenedecanoic acid and DOX in the crosslinked NPs made of fatty acids.

	1-pyrenedecanoic acid	DOX
EE (%)	11.2±1.8	8.2±0.8
LC (%)	1.8±0.2	1.4±0.2
